# *Ab-initio* phasing using nanocrystal shape transforms with incomplete unit cells

**DOI:** 10.1107/S2052252513025530

**Published:** 2013-10-01

**Authors:** Haiguang Liu, Nadia A. Zatsepin, John C. H. Spence

**Affiliations:** a Department of Physics, Arizona State University, PO Box 871504, Tempe, AZ 85287, USA

**Keywords:** shape transform, nanocrystallography, X-ray free electron lasers, phasing

## Abstract

X-ray free electron lasers are used in measuring diffraction patterns from nanocrystals in the ‘diffract-before-destroy’ mode by outrunning radiation damage. The finite-sized nanocrystals provide an opportunity to recover intensity between Bragg spots by removing the modulating function that depends on crystal shape, *i.e.* the transform of the crystal shape. This shape-transform dividing-out scheme for solving the phase problem has been tested using simulated examples with cubic crystals. It provides a phasing method which does not require atomic resolution data, chemical modification to the sample, or modelling based on the protein databases. It is common to find multiple structural units (*e.g.* molecules, in symmetry-related positions) within a single unit cell, therefore incomplete unit cells (*e.g.* one additional molecule) can be observed at surface layers of crystals. In this work, the effects of such incomplete unit cells on the ‘dividing-out’ phasing algorithm are investigated using 2D crystals within the projection approximation. It is found that the incomplete unit cells do not hinder the recovery of the scattering pattern from a single unit cell (after dividing out the shape transforms from data merged from many nanocrystals of different sizes), assuming that certain unit-cell types are preferred. The results also suggest that the dynamic range of the data is a critical issue to be resolved in order to apply the shape transform method practically.

## Abstract 2

The shape transforms of nanocrystals with incomplete unit cells are studied using computer simulations. Structure-factor phases can be retrieved from the molecular transforms after removing the modulating shape transform terms.

## 1. Introduction   

The commissioning of hard X-ray free electron lasers (XFELs) has provided new opportunities for structural biology. It has been demonstrated that ultra-short, extremely intense X-ray pulses from XFELs can yield diffraction patterns from sub-micron crystals in a ‘diffract-before-destroy’ manner, in which radiation damage is outrun, using femto­second exposure times. Single shot diffraction patterns using a beam of micron dimensions have thus been obtained from nanocrystals as small as a dozen or fewer unit cells on a side (Chapman *et al.*, 2011 ▶). This technique, known as serial femtosecond X-ray crystallography (SFX), may provide a new phasing strategy by exploiting the intensity scattered between Bragg spots for very small crystals [for a review of SFX, see Spence *et al.* (2012 ▶)]. Analogous to a finite grating of *N* slits, one sees (*N* − 2) subsidiary maxima in the scattering between Bragg reflections in the experimental patterns. The number of fringes between indexed spots therefore gives the number of unit cells between facets of the nanocrystal along a particular direction. As pointed out by Sayre (Sayre, 1952 ▶, see also Perutz, 1954 ▶, for a related approach), by providing additional intensity samples between Bragg reflections this allows, in principle, a solution of the phase problem *ab initio*. In practice, iterative methods based on this ‘oversampling’ approach have been proven powerful for this purpose (see Spence, 2007 ▶, for a review). For larger crystals these continuous interference fringes can be more simply described as the Fourier transform of the crystal’s external shape envelope, laid down around each reciprocal lattice point, and we will refer them as ‘*shape transforms*’, whose central-maximum width is inversely proportional to nanocrystal size. The Fourier transform of the charge density within one unit cell, which spans all of recip­rocal space, is modulated by these shape transforms around lattice points. For simplicity, we will refer to this unit-cell transform as the ‘*molecular transform*’, despite the possibility of several molecules or *n*-mers per unit cell in protein crystallography.

In previous work we demonstrated by simulation how the complex molecular transform can be recovered if a large number of patterns are available from nanocrystals of different sizes, each showing shape transforms (Spence *et al.*, 2011 ▶). The inverse Fourier transform of this phased molecular transform then provides the required 3D molecular density map in real space. That approach does not phase each pattern separately, since interactive 2D phasing is not practical with the millions of patterns produced at an XFEL using SFX technology, and the final goal is a 3D density map, requiring multiple projections. Our earlier work relied on autoindexing of all patterns to allow 3D merging, then a ‘dividing out’ approach to remove the effects of the shape transforms, by dividing by a separately extracted average shape transform. While successful in simulations, a difficulty arises for crystals which terminate with incomplete unit cells. For example, if the primitive unit cell contains two molecules *A* and *a*, a stacking sequence across one side *AaAaAaAa* (complete cells) will produce an entirely different set of inter-Bragg fringes from the sequence *AaAaAaAaA* (incomplete cells). Such data, merged in 3D, will in addition contain contributions from crystals covering a range of sizes or numbers of complete cells. We must also consider that, in general, the choice of unit cell is not unique in crystallography, so that the addition of molecule *a* on the left of the last stacking sequence produces a nanocrystal which is complete with respect to the new unit cell *aA*. The nanocrystal size distribution function can be obtained, in principle, from the widths of the shape transforms. In this paper we investigate the incomplete unit-cell effect and suggest methods to deal with it.

## 2. Theory and methods   

For plane-polarized, monochromatic incident radiation with wavevector **k**_i_ (|**k**_i_| = 1/λ) and negligible beam divergence, the diffracted amplitude at Δ**k** = **k**_i_ − **k**_o_ produced by a parallelepiped crystallite consisting of *N* = *N*_1_ × *N*_2_ × *N*_3_ unit cells, is given in the kinematic theory (Als-Nielsen & McMorrow, 2011 ▶; Kirian *et al.*, 2010 ▶)
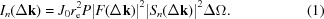
Here, *J*_0_ is the incident photon flux density (photons/pulse/area), *F*(Δ**k**) is the structure factor, *r*_e_ is the electron scattering radius, *P* is a polarization factor and ΔΩ is the solid angle spanned by the detector pixel, and the shape-transform amplitude is
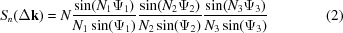
and *F*(Δ**k**) is the structure factor of the unit cell. Here
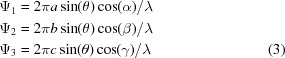
where θ is half the scattering angle, and α, β and γ define the crystal orientation as the angles which the scattering vector Δ**k** makes with the directions of the real-space unit-cell vectors **a**, **b** and **c**. Δ**k** is defined by the position of the detector pixel and X-ray wavelength, and defines a point in reciprocal space where the Ewald sphere intersects the shape transform. An angular integration over the square of the triple product in equation (2) is proportional to *N*_1_ *N*_2_ *N*_3_ and the volume of the crystal. At a Bragg condition, the triple product is equal to *N*_1_^2^ *N*_2_^2^ *N*_3_^2^ and the diffracted intensity is therefore proportional to the square of the number of electrons in the crystal.

In this paper we provide an analysis in the 2D plane normal to the beam. The projection approximation for hard X-ray diffraction holds in the approximation of a flat Ewald surface which passes within the central maximum (measured along the beam direction) of those shape transforms centered on reciprocal lattice points which lie in a plane normal to the beam (the zone axis reflections). Using a parabolic approximation for the sphere, this condition then limits the resolution to *d* > (λ*t*/2)^1/2^, where *t* is the nanocrystal thickness (Spence, 2003 ▶). For nanocrystals with large unit cells, such as the membrane protein Photosystem I, consisting of ten 28-nm unit cells, this would limit resolution using 0.1 nm X-rays to 3.7 nm.

Taking a beam running parallel to the *c* axis (γ = 90°) and orthogonal axes, equation (2) becomes
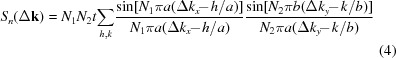
with nanocrystal thickness *t* and scattering angle φ = 2θ ~ λ/*d* for small-angle scattering and |Δ**k**| ~ φ/λ = *q*/2π, where *d* is the crystallographic resolution, the *d*-spacing in Bragg’s law. [The equality of (2) and (4) follows by noting that both functions (in 2D) have the same transform and so must be equal.] The 2D Fourier transform of ψ (Δ**k**, *N*_*i*_) is thus proportional to the low-resolution electron density projected along the beam direction within an orthorhombic supercell of size *N*_1_ **a** × *N*_2_ **b**.

### 2.1. Construction of crystals with partial unit cells at edges   

**Figure 1:**
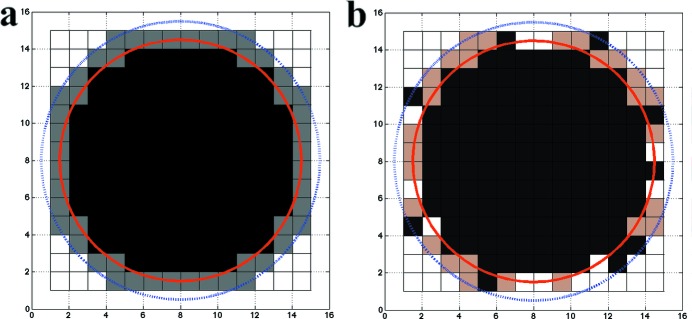
Schematic drawings of crystal boundary (*a*) and an example of crystal composition (*b*). In (*a*), the boundary is defined to be the unit cells in between the two circles (red and blue); the unit cells are colored in grey. In (*b*), the state of boundary unit cells is assigned randomly: full (black), unoccupied (white) and brown (partial). The ratio of three unit-cell states at the boundary is 1:2:1 in (*b*), *i.e.*, 1/3 of the boundary for each state.

A finite square lattice was first constructed in real space and a radius (*R*_0_) was chosen to define the core region occupied by complete unit cells, which were unchanged throughout the simulations. An outer boundary layer is defined by the radius, *R*_1_ = *R*_0_ + *T*_layer_, where *T*_layer_ is the thickness of the boundary in units of unit cells (*T*_layer_ = 1, for these results), as shown in Fig. 1 ▶. There are three possible states for the cells in this boundary layer: fully occupied, partially occupied, or unoccupied. Noting that crystals are usually faceted, the same analysis was carried out for the case of a square core region, and the same conclusions were reached. In each simulation, the probabilities for a boundary unit cell to be in three states are fixed and the actual state is assigned according to these probabilities in a random manner. For a simple 2D case, the unit cell is assumed to be composed of two halves: left and right. For a partial unit cell attached to the left of the crystal center, it has only the right half and *vice versa* for the right side of the crystal. The cases where the unit cell is composed of multiple (>2) subunits are not considered in the current work. Although multiple components per unit cell introduce more complexity, the conclusions found in this simple case should be valid as long as the core region is formed with complete unit cells.

### 2.2. Scattered intensity from a crystal with incomplete unit cells   

Our aim is to extract the unit-cell transform from data described by equation (1), phase it, and so recover an image of the unit cell. We treat the photon flux and polarization factors as constants, and focus on the means to divide out the modulating term 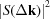 (which depends on crystal shape). In a 3D treatment, we showed (Spence *et al.*, 2011 ▶) that if the diffraction patterns from nanocrystals of different sizes are indexed and merged, then equation (1) may be summed over both crystal orientation and size. Because the scattering factor of a single unit cell is independent of this sum (which is the same for all crystals), it may be brought outside of the sum, and so extracted by dividing the average shape transform into the summed experimental data. For 2D crystals at low resolution where the Ewald sphere is approximately flat, we assume the crystalline monolayers have only one degree of rotational freedom about the axis normal to the layer. Indexing and merging patterns from platelets with a range of sizes, randomly rotated about this axis, would produce the required sum over shape transforms, which we now discuss.

For the case where the crystals are formed from complete unit cells with sharp edges, *i.e.* *N*(*n*) = *N*_1_ × *N*_2_ × *N*_3_, the 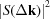 has a simple analytical form as described by equations (2) and (4) above. For irregularly shaped crystals with partial unit cells at the boundary layers, we must sum over all contributing components explicitly, as follows.

The scattering from one unit cell is proportional to the Fourier transform of the electron density ρ(**r**) in the unit cell:
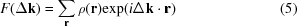
where **r** = **r**(*x*, *y*, *z*) and Δ**k** is the scattering vector. For a 2D crystal, and assuming a flat Ewald surface, this is given by
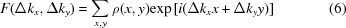
where the summation is over (*x*, *y*) in the single unit cell. For a unit cell located at Δ**R**, the corresponding form factor is
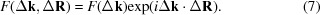
For 2D crystal scattering, we have Δ**R** = (Δ*X*, Δ*Y*) and
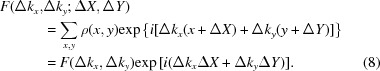
The total scattering form factor of a given crystal can be obtained by summing up the contributions from all unit cells. For the 2D crystal shown in Fig. 1 ▶(*b*), the contribution from each unit cell can be grouped to expedite the calculation of the total Fourier transform.

**Figure 2:**
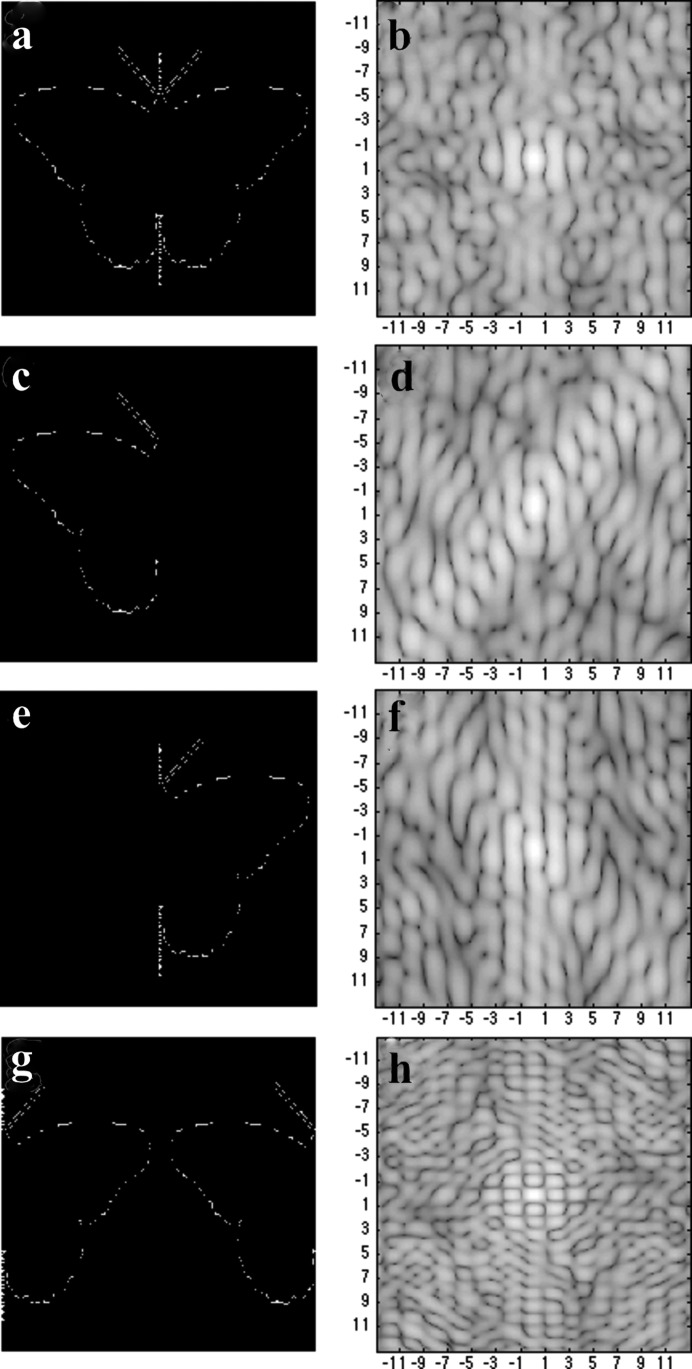
The images used in the simulations. The top row shows the default full unit cell and its scattering pattern. The two middle rows show the left and right half unit cells with their scattering patterns. The bottom row shows the swapped unit cell with its scattering pattern. For the images: the white pixels have density values of 1 and black are 0 (fully transmittive). The intensity is shown on a log-scale using a gray scale map. Axes are marked with the Miller indices.

In our demonstration, the total Fourier transform can be expressed as the sum of three components (see Fig. 2 ▶), 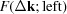, 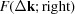 and 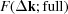, weighted by the summed phase terms,
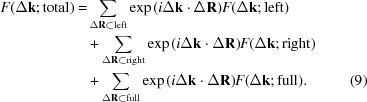
The intensity is obtained as the norm, 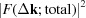.

### 2.3. Recovering the scattering pattern of a single unit cell   

As described in Spence *et al.* (2011 ▶), the scattered intensity from a single unit cell (the ‘molecular transform’) is a smooth function *F*(Δ**K**) with a slow variation which modulates all the Bragg reflections [as in equation (1)], but which may change sign between them. Although the dividing-out approach was originally proposed to study the cases where crystals have complete unit cells and sharp edges, it can also be applied to the present cases where crystals have irregular shapes with partial unit cells at boundaries. In short, first, the indexed intensities are accumulated over the whole dataset; then the average shape transform is obtained by shifting each Bragg spot and its surrounding region to the origin and summing the accumulated scattered intensity for individual pixels in the surrounding region; the final step is to divide this average shape transform into the accumulated intensities for nanocrystals with a range of sizes. This leaves the desiired molecular transform, which is independent of these sums. The recovered scattering pattern is then compared with that of a single full unit cell. The difference is quantified as an *R* factor, defined in a similar way as in crystallography,
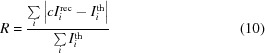
where 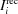 is the recovered scattering intensity and 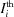 is the theoretical scattering intensity from a single unit cell, both at index *i*, and *c* is the scaling factor.

### 2.4. Phase retrieval   

The hybrid-input-output algorithm (HIO) (Fienup, 1982 ▶) implemented in *Hawk* (Maia *et al.*, 2010 ▶) was used to reconstruct the real space image from the recovered single unit-cell scattering pattern. Initial phases were randomly assigned. The support size is estimated from the autocorrelation function, with an initial support constructed by excluding pixels whose values are below a threshold of 0.1. The HIO feedback parameter, β, was set to 0.9, and reality and positivity were enforced during iterations. The area of the object support was reduced from 10% to 2% of the overall support area within 20 000 steps using the shrink-wrap approach (Marchesini *et al.*, 2003 ▶) and 20 000 extra steps were carried out to ensure convergence.

## 3. Results   

### 3.1. Simulations   

The 2D unit cells used for the simulations are shown in Fig. 2 ▶. A butterfly figure was used as a default binary density ρ(*x*, *y*) for 2D crystals, and the left/right halves are attached to the boundary of the crystals randomly with specified probabilities. Following the crystal packing rules, the left halves are attached to the right side of the crystal to form additional half-unit cells, so their left nearest neighbor is the right half butterfly; the same rule is applied to the right halves. The first three rows show the full and partial unit cells with their corresponding scattering patterns. The fourth row shows an alternative unit cell (whose left and right halves are swapped) and its scattering pattern. The conditions under which the recovered pattern matches the alternative unit cell will be discussed.

### 3.2. Ideal conditions   

We first show simulations for ideal conditions, ignoring instrumental instabilities. We assume an infinite dynamic range for the X-ray detector and unlimited photon flux, which allows accurate intensity measurement within the desired resolution range. These simulation results for the ideal cases establish mathematical foundations for the subsequent work.

**Figure 3:**
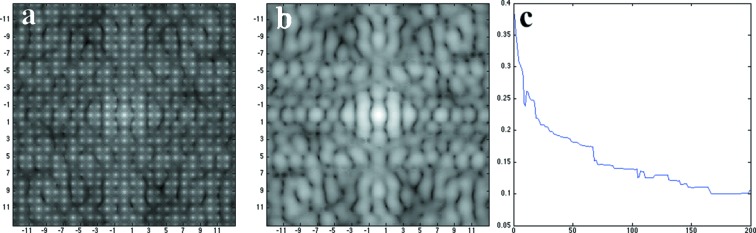
Recovery of the molecular shape transform. (*a*) Accumulated diffraction patterns from 200 crystal diffraction patterns showing Bragg spots; (*b*) The recovered single unit-cell scat­tering pattern (the ‘molecular transform’); (*c*) the *R* factor is progressively reduced as the number of patterns increases. The Miller indices are marked in the patterns, where *k*_max_ = 13.

Following the procedure described in the *Theory and methods* [Sec sec2] section, diffraction patterns were computed from 200 randomly generated crystals with a maximum dimension of 10 unit cells. The core region (see Fig. 1 ▶) is packed with complete unit cells; partial unit cells are randomly placed in 20% of the lattice sites at the boundary layer. Fig. 3 ▶(*a*) shows the accumulated diffraction patterns on a log scale, while Fig. 3 ▶(*b*) shows the recovered molecular transform of the complete unit cell (Figs. 2 ▶ *a* and 2 ▶ *b*). Clearly, the accumulated diffraction pattern has intensities concentrated at Bragg spots and nearby pixels, and the scattering pattern of a single unit cell is recovered after data processing, as Fig. 3 ▶(*b*) is consistent with the theoretical data shown in Fig. 2 ▶(*b*). The *R* factor between the recovered and theoretical molecular transforms de­creased from about 0.4 to 0.1 as the number of patterns approached 200, as shown in Fig. 3 ▶(*c*).

### 3.3. Effects of partial unit cells on boundaries   

**Figure 4:**
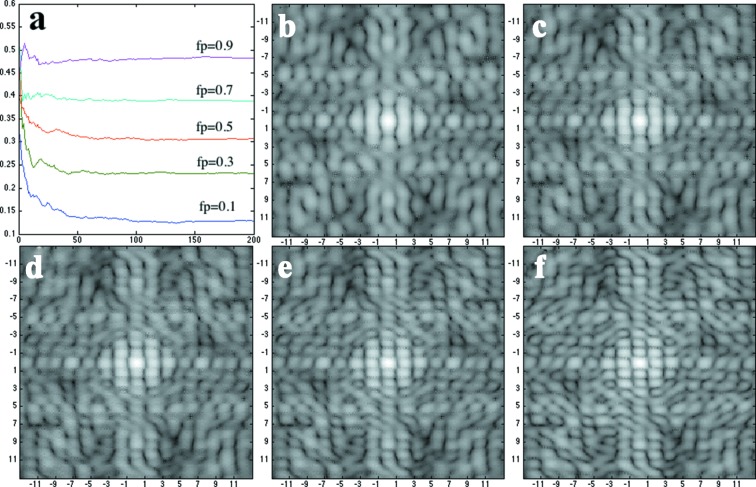
The recovered pattern from crystals with partial unit cells. The fractions of partial unit cell occupied boundary layer are 0.1, 0.3, 0.5, 0.7, 0.9 for (*b*)-(*f*), correspondingly. The *R* factor as a function of the number of diffraction patterns is shown in (*a*) for all five cases with the fraction of partial unit cells indicated. The recovered patterns shown in (*b*) and (*c*) are consistent with the default unit-cell scattering pattern, while the patterns shown in (*e*) and (*f*) are more similar to the scattering pattern of the alternative unit cell. The pattern shown in (*d*) is a superposition of the patterns, as the partial unit cells cover half of the crystal boundary.

As the fraction of partial unit cells at the boundary layer increases, it becomes more difficult to recover the molecular transform. Fig. 4 ▶ shows the *R*-factor progression as more patterns are accumulated. The partial unit cells are placed at boundary layers at five fractions (*f*_*p*_ = 0.1, 0.3, 0.5, 0.7, 0.9), giving five *R*-factor curves. The final recovered molecular transforms for the datasets at five partial unit-cell occupancies are also summarized in Fig. 4 ▶. As *f*_*p*_ gets larger, the *R* factor between recovered patterns and the theoretical pattern of the default unit cell increases, indicating poorer recovery. When *f*_*p*_ is larger than 0.5, however, an interesting phenomenon emerges: the recovered molecular transform becomes more similar to that of the alternative unit cell shown in Fig. 2 ▶. This can be explained by the fact that partial unit cells will form the alternative full unit cell if it is combined with the nearest neighboring halves. As more partial unit cells are placed at the boundary, the number of full alternative unit cells will increase. When partial unit cells cover half of the boundary, the two types of full unit cells are equally weighted; therefore the recovered scattering pattern is a superposition of two scattering patterns (Fig.4*d* is the superposition of patterns shown in Fig. 2 ▶ *b* and 2*h*). As the partial unit cells cover more than half of the boundary layer, the alternative unit cell becomes dominant, and the recovered molecular transform becomes more consistent with the pattern of the alternative unit cell (Figs. 4*e* and 4*f* show features of Fig. 2*h*).

**Figure 5:**
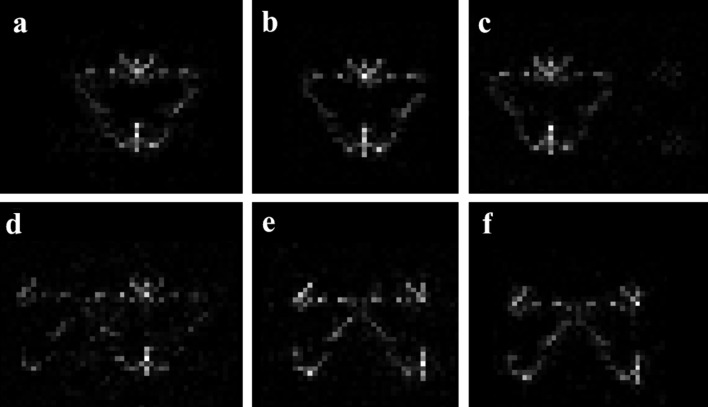
The real space image construction *via* the *ab initio* phase retrieval (HIO) method. The same control parameters were applied for all six cases (see *Theory and methods* [Sec sec2] for details). The reconstructed object from theoretical data is shown in (*a*), and (*b*)–(*f*) are the reconstructions from the recovered patterns shown in Figs. 4 ▶(*b*)–(*f*), respectively.

The real space images were constructed from the recovered oversampled patterns using an *ab initio* phase retrieval approach described in the *Theory and methods* [Sec sec2] section. The reconstructed images are shown in Fig. 5 ▶, including the image reconstructed from theoretical data (Fig. 2 ▶ *b*). The results are consistent with the observations of scattering patterns. Depending on the fraction of the boundary layer that is occupied by partial unit cells, the real space images reconstructed are either the default unit cell (Figs. 5*b*,*c*) or the alternative unit cell (Figs. 5*e*,*f*), or the superposed image (Fig. 5 ▶ *d*). Compared with the image in Fig. 5 ▶(*a*), we can see that the recovered patterns contain sufficient information for a faithful model reconstruction. Even for the worst scenario where the recovered pattern is a superposition of the two distinct unit-cell transforms, the reconstructed real space image reveals important features of the original object.

**Figure 6:**
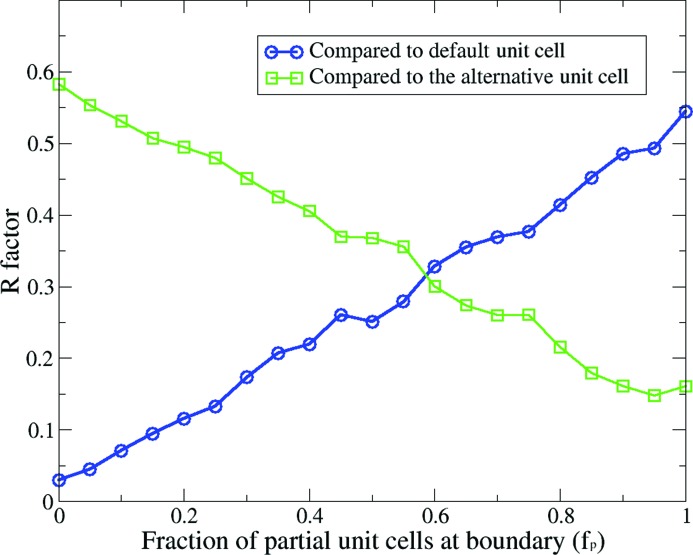
The *R* factors between the recovered patterns and the default (or the alternative) unit-cell transforms [equation (10)]. The trend clearly indicates that the recovered patterns drift away from the default unit cell and toward the alternative unit cell, as larger fractions of crystal boundary are occupied by partial unit cells.

The similarities between the recovered patterns and the transform of the default unit cell were quantified using *R* factors. Likewise, the *R* factors between recovered patterns and the alternative unit-cell transform are also calculated. The results for a range of partial unit-cell fractions are summarized in Fig. 6 ▶. As expected, the *R* factors are *anti*-correlated with the partial unit-cell fractions (*f*_*p*_), showing a clear shift from one unit-cell type to the other. This poses a serious problem when handling real experimental data, where we may not have any control over partial unit-cell coverage at the boundaries. It makes data processing very challenging as we would need to classify the patterns into groups from which we can recover distinct patterns from certain types of unit cells. On the positive side, biological molecules often have preferred interacting surfaces, along which the molecular binding interaction is stronger. As a consequence, some types of unit cells should be favored over others. For instance, the default unit cell in Fig. 2 ▶(*a*) might be preferred because there are more contacts between the two subunits, compared with the alternative unit cell. If this is the case, then the transform of the favored unit cell should be dominant and will be the one that is recovered using this approach. To conclude, the described approach will work as long as the unit cells are not equally favored when forming crystals, since the choice of unit cell is not unique.

### 3.4. Crystal size variations   

**Figure 7:**
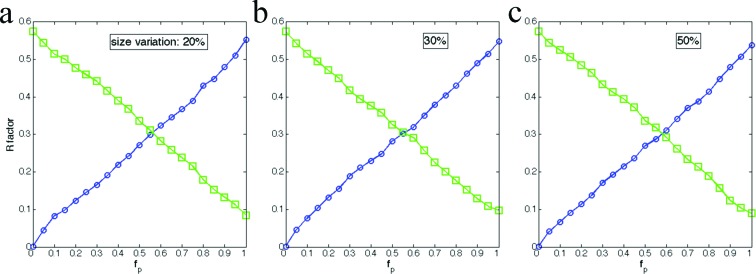
Crystal size variations do not affect molecular transform recovery. The crystal sizes vary 20%, 30% and 50% around the mean (10 unit cells). The variations in crystal sizes do not hinder the recovered pattern quality, indicated by the *R* factors compared with the theoretical patterns from default unit cell (circles) or alternative unit cell (squares). The axes are defined as in Fig. 6 ▶. Green line: original unit cell; blue line: alternate unit cell.

Crystal size variations cannot be avoided in reality. By generating crystals with different sizes, the size variation effects were investigated in this study. The default diameter of crystals was set to be 10 unit cells, which is the same as in the simulations for the ideal cases. The individual crystal diameter was randomly generated to allow a maximum of 2, 3 or 5 unit-cell variations. For each crystal, the partial unit cells were placed at the boundary according to the desired fractions of boundary occupied by partial unit cells. The *R* factors are plotted in Fig. 7 ▶. It is clear that the crystal size variations do not reduce the recovered pattern quality, compared with the ideal case where the crystal core had a fixed size and only the partial unit-cell fraction of the boundary was varied. In summary, the crystal size variations do not reduce the recovered scattering pattern quality.

### 3.5. Limitations due to the dynamic range of detector   

**Figure 8:**
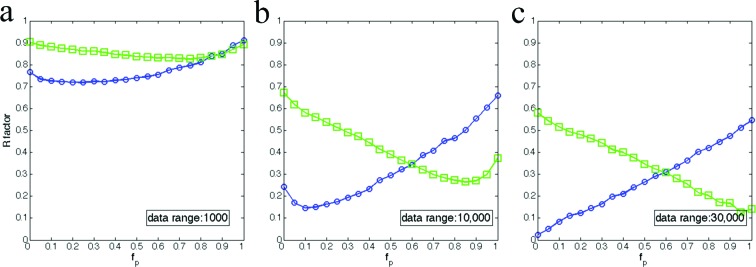
Measurable data range is critical to recover the molecular transforms. The data ranges are indicated in each plot. The partial unit cells cover 20% of the boundary layer, *i.e.* *f*_*p*_ = 0.2. The corresponding recovered molecular transforms are shown in Fig. 9 ▶. The axes are defined as in Fig. 6 ▶.

In real experiments, the photon flux and detector’s dynamic range together set limits on the data range that can be collected. For example, if the scattered number of photons is very small even at low angles because of low photon flux, the maximum number of photons that can be recorded is limited. The dynamic range of detectors also imposes limitations on the intensity range. Typical detectors used in X-ray crystallography experiments have a dynamic range between 10^3^ and 10^4^ photons/pixel. Here, our purpose is to study the consequences of a limitation on the data range due to these two factors. We assume that the photon flux and detector dynamic ranges are optimally matched to allow us to measure the highest intensity at the upper limit, and the lower limit sets the measurable weakest intensity. In simulations, this means the data will be scaled linearly such that the maximum intensity is equal to the data range, and all the intensity values smaller than one will be zero. Thus, the data range actually is a parameter that controls the lowest intensity that can be measured, and therefore, we refer this to as the ‘measurable data range’. It is clear that the measurable data range is critical for the recovery of molecular transforms. The *R*-factor plots are summarized in Fig. 8 ▶ for three different measurable data ranges. For a measurable data range of 10^3^, it is almost impossible to recover an accurate scattering pattern for the simulated 2D crystals. For data with an intensity range of 10^4^, the recovered patterns are reasonably accurate, and, furthermore, the recovery accuracy becomes comparable to the ideal scenario when the data range gets to 3 × 10^4^. The desired data range depends on the samples, especially the number of unit cells in the crystal that determines the modulating function, the shape transform.

**Figure 9:**
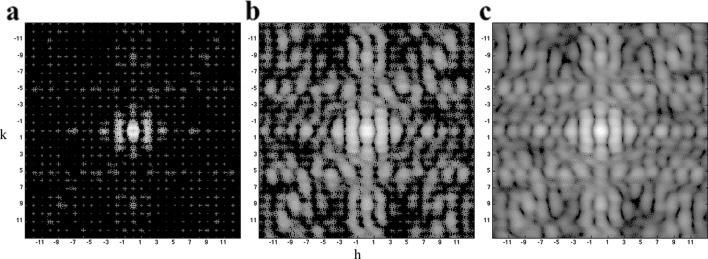
The recovered molecular transforms at different data ranges. The partial unit cells cover 20% of the boundary layer, *i.e.* *f*_*p*_ = 0.2. The data ranges are 10^3^, 10^4^ and 3 × 10^4^ for (*a*), (*b*) and (*c*), correspondingly. For a small data range, the weak scattering in inter-Bragg regions is not recovered (*a*); more intensities are recovered at medium data range [10^4^, in (*b*)]; and, eventually, the recovered pattern is comparable to the results in the ideal case when the measurable data range gets to 3 × 10^4^ (*c*).

Due to the interference effects, the strong scattering is near the Bragg spots while weak scattering happens nearly midway between Bragg spots. As a result, it is difficult to recover the scattering pattern at high resolution and for the regions that are in the middle of Bragg spots when the dynamic range is small and tuned to measure high intensities without saturating the pixel (Fig. 9 ▶).

### 3.6. Crystal shape effects   

**Figure 10:**
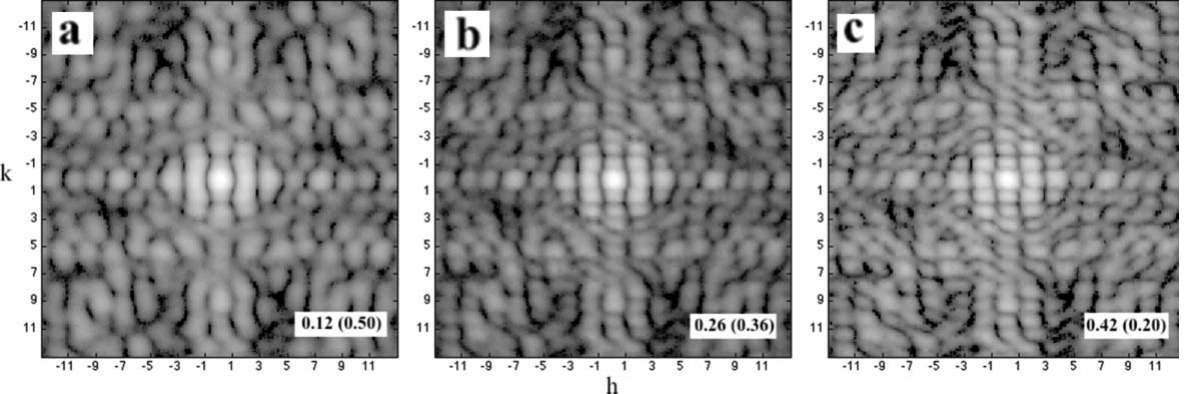
Crystal shape variations do not reduce the recovered molecular transform qualities. The fraction of boundary that is covered by partial unit cell is the determining factor for the recovered unit-cell patterns. The fractions are 0.2, 0.5, 0.8 for (*a*), (*b*), (*c*), correspondingly. The numbers shown in the lower right corners are the *R* factors compared with the theoretical unit-cell transform, relative to the default unit cell; and the values in parentheses are the *R* factors compared with the alternative unit-cell transform (see Fig. 2 ▶ *h*).

The crystal can form various shapes, apart from the rounded shape used here for the case of 2D crystals. In general it will be facetted and often express the point group symmetry of the crystal, or be determined by the Wulff construction if in equilibrium (Sekerka, 2005 ▶). Here, by generating oval shapes with different radii and adding full and partial unit cells to the boundary, crystal shape effects were investigated under more realistic simulation conditions: 3 × 10^4^ dynamic range, mean radius of 5 unit cells with up to 2 unit-cell variation along the major and minor axes (40% variation), with Poisson noise added to the patterns. The recovered patterns are shown in Fig. 10 ▶, at *f*_*p*_ = 0.2, 0.5, 0.8. Surprisingly, crystal shape and size variation did not diminish the accuracy of the recovered molecular transform. The *R* factors corresponding to Fig. 10 ▶ are almost identical to those in Fig. 8 ▶(*c*), which was based on the same parameters without crystal size or shape variation.

### 3.7. Classification of nanocrystal terminations   

**Figure 11:**
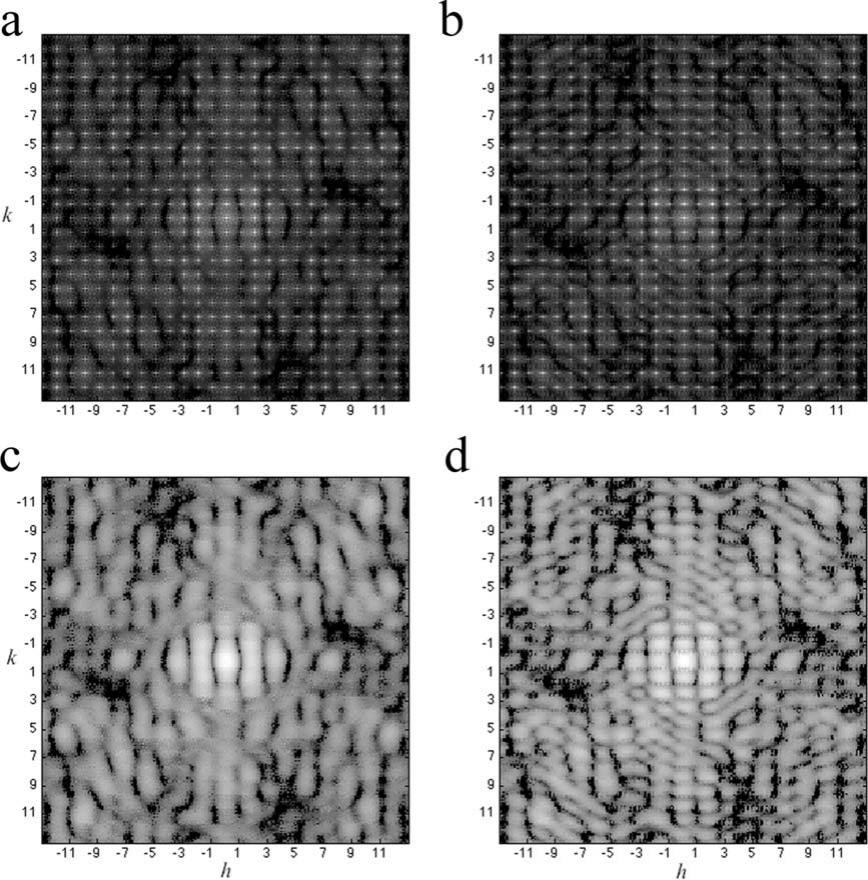
Classification of diffraction patterns from crystals with different numbers of partial unit cells at the boundary. Using the expectation maximization algorithm, the two classes of diffraction patterns are separated and the accumulated diffraction patterns are shown in (*a*), (*b*) for each class; the corresponding recovered molecular transforms are shown in (*c*), (*d*).

Under certain circumstances, for example, when the variation of the fraction of partial unit cells at the boundary is narrowly distributed, pre-processing the diffraction patterns and classifying them based on their similarities may allow recovery of molecular transforms for multiple unit-cell types. We tested the expectation maximization (EM) algorithm (Loh & Elser, 2009 ▶) for this purpose. This has been developed for single particle scattering orientation recovery and data assembly. By modifying the algorithm to handle multiple model cases, the EM algorithm can be applied to separate the diffraction patterns of 2D crystals into different classes. The distance between patterns (a measure of their similarity) was measured using *R* factors. In each simulation, 200 patterns were collected from mixtures of crystals with partial unit cells, covering a varying fraction of the 2D crystal boundary layer, where 100 patterns resulted from crystals with a mean *f*_*p*_ = 0.2 and the other 100 with a mean of *f*_*p*_ = 0.8. To classify the 200 simulated patterns using the EM algorithm, two patterns were generated using random intensities to seed merged patterns and start the iterative classification. Under the EM algorithm, individual simulated patterns were assigned to one of the two classes based on the distance (measured by *R* factor) between the patterns and the seeding pattern. After class assignments for each simulated pattern, two newly merged patterns were computed by summing the patterns belonging to the class. These two new merged patterns then served as the references for the next iteration. Once the classification converged, the EM algorithm was terminated, and the 200 patterns were separated into two classes. All the simulated patterns came from crystals with the same fixed core region and a varying boundary layer, and the data range was set to be 3 × 10^4^ with Poisson noise. In this simulation, 195 patterns were correctly assigned to the correct groups and only 5 were wrongly classified. The recovered molecular transforms from the classified patterns are shown in Fig. 11 ▶. Although we had to simplify the problem by assuming a fixed core region, such an approach offers a possible solution for extracting information from a large ensemble of patterns resulting from unclassified crystals.

## 4. Discussion and conclusions   

Serial femtosecond nanocrystallography (SFX) using XFEL pulses not only allows the collection of diffraction data at Bragg spots, but also provides opportunities for phasing by exploiting the scattering between Bragg spots. Using this ‘dividing-out’ approach, the oversampled scattering intensities for a single unit cell can be recovered, and phase retrieval can be accomplished subsequently *via ab initio* phasing algorithms. Unlike the ‘direct methods’ numerical approach used in protein crystallography, this approach does not require atomic resolution data; unlike the single isomorphous replacement (SIR) approach, it does not required chemical modification to the sample; and unlike the multi-wavelength anomalous diffraction (MAD) method, it does not require the operation of an X-ray source at specific energies.

It has been a concern that the incomplete unit cells at the surfaces of crystals introduce signals that cannot be decoupled from the scattering pattern of a single unit cell (see *Acknowledgements*). In this work, using a simplified 2D crystal system, we demonstrated that molecular transform recovery is possible even in the presence of such incomplete unit cells. In our simplified model, the unit cell was composed of two subunits, thus the recovered molecular transform could be a linear combination of the molecular transforms of the default and alternative unit cells, depending on the fraction of the boundary that is covered by partial unit cells. In a real crystal, certain types of unit cells are preferred over others, due to the different binding affinity between subunits. This provides a chance to recover the molecular transform of the preferred unit cell. We found that the measurable data range is the main limiting factor that determines the recovered pattern quality. In order to get accurate molecular transforms, the experiments must record data at inter-Bragg regions. The other relevant issue concerns the achievable resolution. Since the intensity decrease is proportional to *q* ^−4^ for molecular transforms, the measurable intensity range sets a limit on the data resolution. The shape transform imposes a local variation on intensities by multiplying the modulation function with the molecular transform around each Bragg spot. This modulation function sets the limits of measurable intensity around the vicinity of the Bragg spots, as observed in Fig. 9 ▶. Based on the Shannon theorem, only a single additional intensity sample is ideally needed midway between Bragg spots. In the presence of noise, additional sampling points between neighboring Bragg spots improve *ab initio* phase retrieval, to build an electron density map. In these simulations, we sampled 8 points between Bragg spots, and the *R* factor was calculated based on all non-zero intensities. As mentioned above, the intensity between Bragg spots decays rapidly as the scattering vector **k** moves away from Bragg spots, and the requirement on the data range can be relaxed if fewer points between Bragg spots are needed (*i.e.* the pixels that are not too far away from Bragg spots). An even number of samples between Bragg spots avoids the weakest scattering at midpoint. Nevertheless, a larger dynamic range and increased photon flux would allow us to record intensities at more pixels, and to recover scattered intensities over a larger fraction of the intensity space.

In summary, it is possible to extract the molecular transform of a single unit cell even in the presence of partial unit cells, provided a unique, complete unit cell can be defined that is dominant over the whole crystal. The variation of crystal size does not hinder the accuracy of molecular transform recovery using the dividing-out approach. The most critical factor is the measurable data range that is determined by sample, X-ray brilliance and detector: the requirement is that the measurable data range should be large enough to allow the recovery of the weak inter-Bragg scattering, in order to retrieve the phases using this oversampling approach.
